# REOPENING UNDER COVID-19: THE IMPACT OF REOPENING SOCIETY ON OLDER ASIAN AMERICAN’S DEPRESSIVE SYMPTOMS

**DOI:** 10.1093/geroni/igac059.1260

**Published:** 2022-12-20

**Authors:** Yifan Lou, Ethan Siu Leung Cheung, Jinyu Liu, Bei Wu

**Affiliations:** Columbia University, New York, New York, United States; Columbia University, New York, New York, United States; Columbia University, New York City, New York, United States; New York University, New York, New York, United States

## Abstract

**Background:**

The lockdown due to COVID-19 has influenced individuals’ lives in many aspects. Yet, the impact of reopening under an ongoing pandemic is understudied. This study aims to investigate the impact of reopening policy on older Asian Americans’ depressive symptoms and whether the impact varies by their sociodemographic characteristics.Method: We used interview data collected from 519 Chinese and Korean aged 60 and older in New York City between 5/23/2021 to 7/30/2021. Interrupted time series model was used to test whether there are significant level and slope changes in depressive symptoms (PHQ-9 scale) before and after the reopening on 7/1/2021 in NYC. We then ran the models in stratified sample by gender, education, income, self-reported health, and social connectedness through living arrangements, use of technology, and social interactions.

**Results:**

Older Asians’ depression increased immediately following the reopening (ß=1.52, p< 0.05), and then slowly decreased then after (ß=-0.12, p< 0.001). A decrease in depression following reopening was significantly associated with the male gender, good health, higher income, living alone, having received or provided social support, daily texting, and no engagement in the discussions related to COVID-19 in social media. Discussions: While reopening may have long-term benefits on mental health, older Asians were anxious about their safety at the beginning of reopening under an ongoing pandemic. Older adults with worse health, lower SES, and limited social connectedness struggled to adjust to “back-to-normal” life. We discussed research, policy, and practice implications to support these disadvantaged older adults after reopening.

